# Effect of flow rate ratio and positioning on a lighthouse tip ECMO return cannula

**DOI:** 10.1007/s10237-023-01741-2

**Published:** 2023-07-16

**Authors:** Francesco Fiusco, Julien Lemétayer, Lars Mikael Broman, Lisa Prahl Wittberg

**Affiliations:** 1 FLOW, Department of Engineering Mechanics, KTH Royal Institute of Technology, Stockholm, Sweden; 2 ECMO Centre Karolinska, Pediatric Perioperative Medicine and Intensive Care, Karolinska University Hospital, Stockholm, Sweden; 3 Department of Physiology and Pharmacology, Karolinska Institutet, Stockholm, Sweden

**Keywords:** ECMO, CFD, Return cannula, Hemolysis, PIV, Recirculation, POD

## Abstract

Extracorporeal membrane oxygenation is a life-saving support therapy in the case of cardiopulmonary refractory failure. Its use is associated to complications due to the presence of artificial surfaces and supraphysiological stress conditions. Thus, knowledge of the fluid structures associated to each component can give insight into sources of blood damage. In this study, an experimentally validated numerical study of a conventional lighthouse tip cannula in return configuration was carried out to characterize the flow structures using water or a Newtonian blood analog with different flow rate ratios and cannula positioning and their influence on hemolysis. The results showed that strong shear layers developed where the jets from the side holes met the co-flow. Stationary backflow regions at the vessel wall were also present downstream of the cannula. In the tilted case, the recirculation was much more pronounced on the wide side and almost absent on the narrow side. Small vortical backflow structures developed at the side holes which behaved like obstacles to the co-flow, creating pairs of counter-rotating vortices, which induced locally higher risk of hemolysis. However, global hemolysis index did not show significant deviations. Across the examined flow rate ratios, the holes on the narrow side consistently reinfused a larger fraction of fluid. A radial force developed in the tilted case in a direction so as to recenter the cannula in the vessel.

**Supplementary Information:**

The online version contains supplementary material available at 10.1007/s10237-023-01741-2.

## Introduction

To treat patients with severe lung and/or heart failure, extracorporeal membrane oxygenation (ECMO) can be used as a life-saving support therapy. The ECMO circuit is composed of a pump (centrifugal or roller), a membrane oxygenator with heat exchanger, cannulae for drainage and reinfusion of blood to the body, and tubing with connectors. Due to the occurrence of non-physiological flow conditions in the circuit, the use of ECMO is associated with damage of blood components. This induces a higher risk for hemolysis and thrombosis (Olson et al. 2021; Balle et al. 2018). The use of ECMO requires management of anticoagulation, usually performed by intravenous administration of unfractionated heparin, or direct thrombin inhibitors. This effectively balances the risk of thromboembolic complications with that of bleeding, the most common causes of death and morbidity during ECMO (Fletcher-Sandersjöö et al 2017; Iacobelli et al. 2021).

Blood damage during ECMO is mainly due to mechanical stresses arising in the flow field associated to the different components (Balle et al. 2018; Casa and Ku 2017; Faghih and Sharp 2019), motivating further understanding of ECMO circuit components hemodynamics to assess and mitigate undesired flow phenomena.

A plethora of cannula designs and sizes for different cases are used in clinical setting (Broman et al. 2019a, b) and adverse events due to cannula/cannulation have been explored as by Litwak et al. (2008); Haymet et al. (2021). From an engineering perspective, the flow field in cannulated vessels shows features resembling some canonical flow scenarios. In the case of drainage configuration, cannulae with side holes (e.g., with a *lighthouse tip* (single stage)) exhibit a *jet in crossflow* type of behavior, characterized by strong shear layers in the drainage area (Fiusco et al. 2022). In the return cannula flow, using a blunt cannula, a *confined jet* surrounded by a co-flow develops, as shown by Lemétayer et al. (2020, 2021). This case has been widely studied in the literature, albeit relatively few groups documented investigations on highly confined jets (i.e., with diameter ratio between outer vessel and nozzle $D/d \le 6$), e.g., (Zhdanov et al. 2006). It has been shown that for certain confinements and flow rate ratios, a backflow develops along the walls of the outer vessel close to the jet exit. Henzler (1978) showed that the backflow appears when the condition given by $D/d > (G + \dot{V}_\text {D}/\dot{V})d$ is fulfilled, with *D* and *d* being the inner diameters of the outer and inner cylinders and $\dot{V}_\text {D}$ and $\dot{V}_\text {d}$ being their respective flow rates. *G* is a constant being close to 1.0 for equally dense media.

To characterize and minimize the risk of flow-induced blood trauma in cannula flows, more detailed knowledge is required regarding the fluid dynamics of return cannulae in the presence of side holes, such as when lighthouse tip designs are used. In the case of ECMO, the position and angle of the cannula in the vessel may not be precisely known and is influenced by a variety of factors, since flexible cannulae are used. Thus, quantitative knowledge of the effects of lateral positioning and flow rate ratio on the flow structures, forces and reinfusion characteristics is useful to quantify the influence of design choices and insertion angle. Whether this also plays a role on blood damage level gives also useful insight.

The aim of this study was to characterize the flow structure dynamics during different flow rates and positions for a return lighthouse tip cannula. Moreover, the effect of different flow conditions and the impact on blood damage levels (quantified by hemolytic index) was assessed. The study was carried out using Computational fluid dynamics (CFD) simulations. The numerical results were validated by experimental data obtained from particle image velocimetry (PIV) measurements.

## Methods

**Fig. 1:**
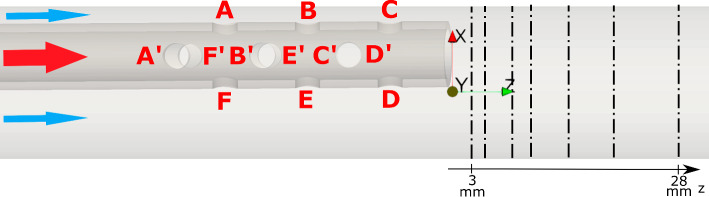
Considered cannula geometry in tilted position. The cannula and co-flow inlets are placed on the far left of the domain (as indicated in the figure—red is cannula flow, blue is annular co-flow), whereas the outlet is placed on the right. The side holes are labeled A to F, with prime symbols distinguishing the out-of-plane holes A, F, A’ and F’ represent the most proximal (upstream) side holes. The area between holes A, B and C and the vessel is referred to as "narrow side", whereas the area facing holes F, E and D is called "wide side". The dash-dot lines indicate the planes used for validation (cfr. Sect. 3.1)

The considered flow scenario mimicked a lighthouse tip ECMO cannula in return configuration. In particular, a veno-venous (VV) ECMO was considered, in which blood is returned in either the inferior or superior vena cava, aligning cannula flow with native vessel flow direction. The geometry is shown in Fig. 1, where the outer tube represented the vessel and had an internal diameter of 18.3 mm. The (glass replica) cannula had an outer diameter of 8 mm with a wall thickness of 1 mm and a length of 500 mm. Twelve circular side holes with 3 mm diameter were present on the surface, arranged in a shifted manner on each quadrant. The cannula was placed coaxially with the vessel and then tilted 3.4 mm at the tip with respect to the centerline of the vessel. Considering the length of the cannula, the tilting angle was computed to $0.39\,^\circ$, allowing for the cannula to be considered as *shifted* with respect to the centerline of the vessel. When placed in a vessel, an ECMO cannula, which is flexible, is unlikely to remain in a constant position. The two positions investigated in this study were chosen to capture the most different flow dynamics that may develop in such flow: a cannula perfectly aligned with the vessel and a cannula located closely to the vessel wall. Figure 1 includes the coordinate system, with the *z* axis (*w* velocity component) oriented in the streamwise direction, the *x* axis (*u* velocity) placed normally and the *y* axis (*v* velocity component) completing the Cartesian system.

The considered cases are reported in Table 1. Cases 1 and 2 were used to assess the effect of cannula positioning on the resulting flow field, using water in order to compare with available experimental particle image velocimetry (PIV) data obtained as explained in Sect. 2.1. Time-averaged velocity profiles obtained from numerical and experimental setups were compared for this purpose. Cases 3, 4 and 5 used a Newtonian analog of blood with a viscosity of $\mu =3.0\,\text {mPa}$ s, corresponding to a hematocrit of around $20\%$. The co-flow ($Q_v$) was scaled to preserve the same Reynolds number as the water case with the larger viscosity, with the Reynolds number being defined as:

1 $$\begin{aligned} \text {Re} = \dfrac{\rho Ud}{\mu } \end{aligned}$$

where $\rho$ represents the density, *U* a reference velocity (given by the flow rate divided by the cross-sectional area) and $\mu$ the dynamic viscosity. Different cannula flow rates ($Q_\text {c}$) were considered to assess their effect on the flow structures; in particular, $Q_\text {c}=6.0$ L/min had a flow rate ratio closer to clinical practice, while preserving the dynamic similarity with respect to the simulations for water (Cases 1 and 2).

**Table 1:** Considered flow cases

Case	Cannula flow (L/min)	Co-flow (L/min)	Fluid
1 (centered)	2.6	1.3	Water
2 (tilted)	2.6	1.3	Water
3 (tilted)	3.9	3.9	Newtonian analog
4 (tilted)	6.0	3.9	Newtonian analog
5 (tilted)	7.8	3.9	Newtonian analog

### Experimental method

The experimental setup was similar to the one used by Lemétayer et al. (2020, 2021). Two parallel circuits were used to generate the cannula flow and co-flow, with the cannula circuit being driven by a centrifugal blood pump. The two co-axial tubes were enclosed in a box filled with water to reduce optical interference due to curved surfaces.

The PIV system was composed of a high-speed double frame CCD camera by Dantec with a resolution of $1920\times 1200$ pixel and 12-bit pixel depth mounted on a Nikkor lens (105 mm, $f/d_\text {max}=1.8$) and a high-speed Nd:YAG laser beaming at 532 nm to generate a 1 mm thick sheet through the cannula centerline. A 20 mm extension ring was applied to reach a magnification factor of 31 pix/mm. Solid zirconium oxide particles with a diameter of $5\,\upmu$m were used as tracers.

The interrogation window over which data was collected was a longitudinal plane along the cannula axis extending 60 mm downstream of the cannula tip. A total of 1000 couple images were sampled with a frequency of 200 Hz.

The velocity field was generated from the images with an in-house code developed by Lecordier and Trinite (2004).

### Numerical setup

All cases were simulated using the commercial CFD software Star-CCM+ (v. 17.02.007). The 3D incompressible Navier–Stokes equations were solved using an implicit large eddy simulation (LES) approach, i.e., no sub-grid scale (SGS) model. The computational mesh comprised 10.7 million cells and 10.2 million cells for the centered and tilted case, respectively. Details about meshing strategy are reported in Online Resource 1. The timestep was fixed and equal to 0.0001 s. The cannula flow-through time was estimated to be 0.87 s, resulting in that the used timestep was equivalent to 0.00011 cannula flow through times. As for boundary conditions, plug profiles were applied to the co-flow and cannula inlets (making the co-flow inlet annular), the walls were treated with a no-slip condition and the outlet was a simple pressure outlet, placed 500 mm away from the tip of the cannula to avoid effects on the flow field (and to match the experimental setup). Data including velocity, pressure, vorticity and shear rate fields were exported on a longitudinal plane in the center of the cannula and in a cylindrical region around the reinfusion zone with a sampling rate of 100 Hz. All data was averaged over 5 s, i.e., 5.75 cannula flow-through times.

The hemolysis index (HI) was computed together with the flow field. A power-law formulation was employed:

2 $$\begin{aligned} H = C\tau ^\alpha t^\beta \end{aligned}$$

where *H* is the hemolysis index (fraction of plasma-free hemoglobin compared to the total hemoglobin), $\tau$ is a scalar form of the shear stress (in this case, the Frobenius norm was used) and *t* is exposure time. $C,\alpha ,\beta$ are model constants. To allow better integration with the CFD solver, the model was casted as a transport equation following Yu et al. (2017):

3 $$\begin{aligned} \dfrac{\partial H_L}{\partial t} + (\textbf{u}\cdot \nabla )H_L = C^{\frac{1}{\beta }}\tau ^{\frac{\alpha }{\beta }}(1-H_L) \end{aligned}$$

where $H_L=H^{\frac{1}{\beta }}$. The hemolysis value corresponding to the whole domain was collected at the outlet by considering a mass-flow average, applying the set of model constants provided by Heuser and Opitz (1980).

## Results

### Experimental validation

**Fig. 2:**
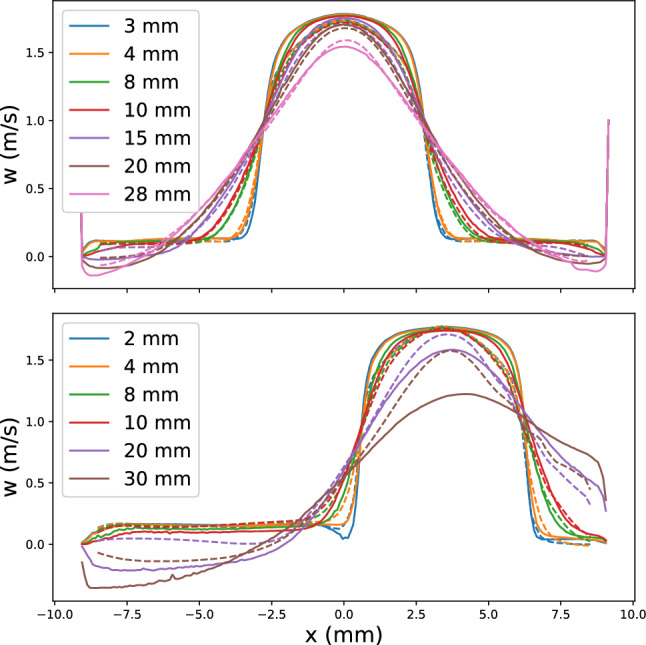
Time-averaged streamwise velocity profiles sampled downstream of the cannula for Cases 1 (top) and 2 (bottom). The solid lines represent data gathered from the simulation, the dashed lines depict experimental data. The values in the legend indicate the distances from the cannula tip at which the velocity data was acquired, Fig. 1

**Fig. 3:**
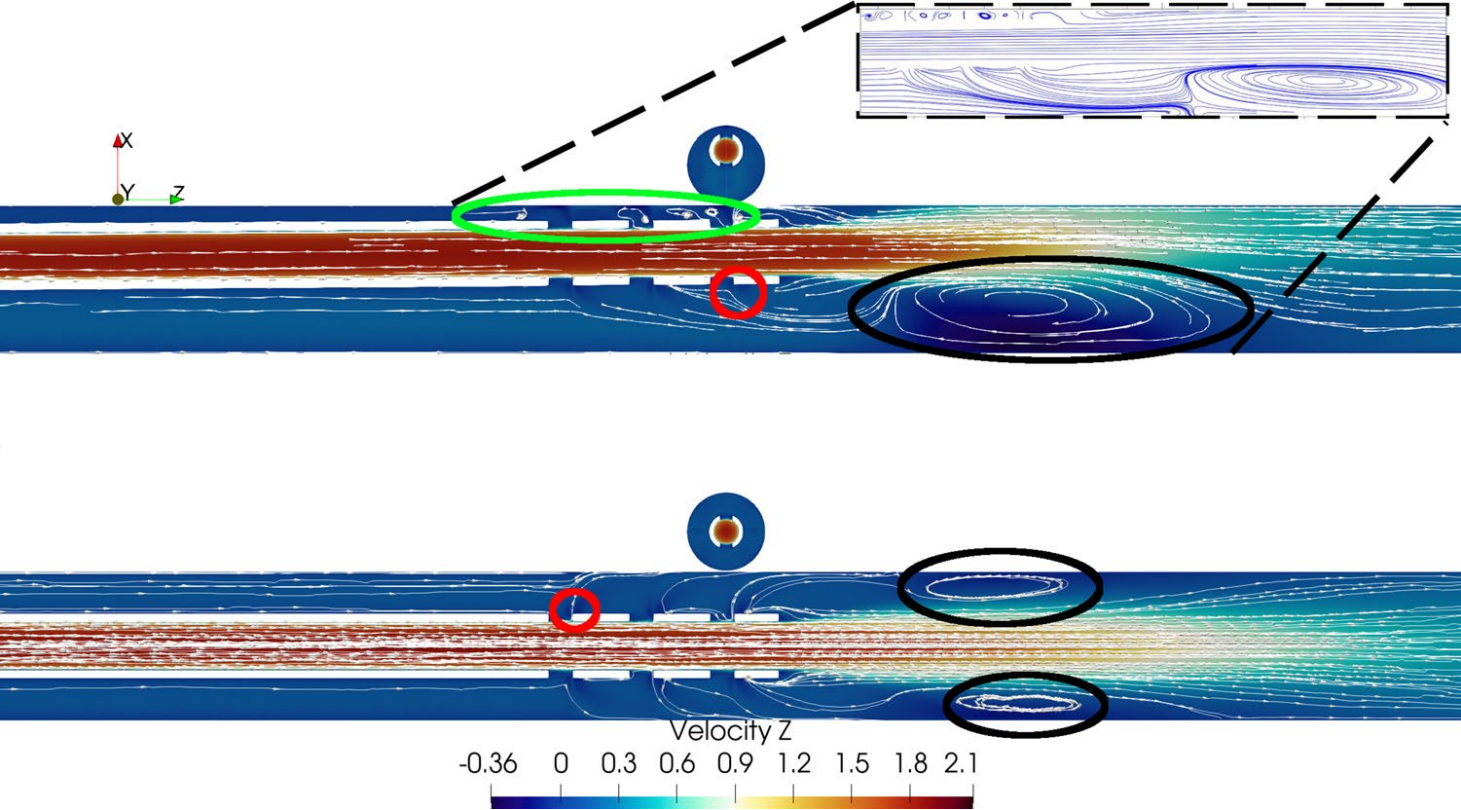
Baseline flow field for Cases 1 and 2 (bottom and top respectively). Downstream of the cannula tip recirculation zones were observed at the vessel walls, in both the centered and tilted cases (black circles). In Case 1 these zones were symmetrically present on both sides, whereas in Case 2 recirculation was more pronounced on the wide side and almost absent on the narrow. The streamlines also show the development of shear layers where the jet coming from the side holes met the co-flow (red circles). On the narrow side of Case 2, the shear layers rolled up in a vortical recirculation region (green ellipse), which was also observed experimentally (insert in black dashed lines, obtained from PIV data; notice the recirculation areas in the top left)

Figure 2 shows the time-averaged streamwise velocity profiles downstream of the cannula according to the planes defined in Fig. 1 for Cases 1 and 2. The results show good agreement between experiments and simulations for Case 1, while discrepancies up to 20% could be observed for the tilted case more than 3 cm downstream of the cannula tip, possibly due to difference in geometry when tilting the cannula. Moreover, the flow dynamics showed a transitional behavior that presents some challenges in numerical characterization. In particular, larger momentum diffusion was observed in the numerical data further downstream the cannula tip. The use of a wall-adapted eddy viscosity (WALE) subgrid-scale model led to better agreement in the region further from the cannula tip (Online resource 1, Figures S1.5, S1.6). This highlights the carefulness required in modeling this highly unsteady flow. However, as the region of interest in this study was around the side holes, the results of the implicit LES were considered adequate, as also shown in Fig. 3.

### Influence of cannula positioning

Time-averaged streamwise velocities for Cases 1 and 2 are shown in Fig. 3.

A few important features can be highlighted:

- A backflow along the vessel wall was present in both cases, as previously reported in the literature (Lemétayer et al. 2020, 2021). In both cases, the backflow was stationary (see Online Resource 2). In the tilted case, the recirculation zone was more pronounced, as also demonstrated by the streamlines superimposed to the velocity field and highlighted with black circles.
- Strong shear layers developing along the side holes, clearly marked in the centered case and visible in the wide side of the tilted case, indicated with red circles.
- On the narrow side of the tilted case, small recirculation zones with a vortical behavior were found at each side hole (green ellipse). Their presence was also confirmed experimentally (Fig. 3, insert).

**Fig. 4:**
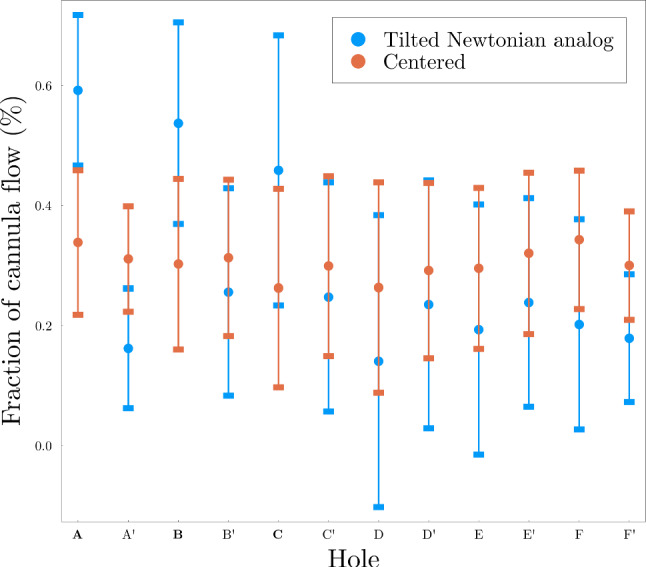
Distribution of reinfused flow among the side holes (in percentage of the total reinfused flow rate), averaged over 5 s. The vertical bars represent the standard deviation of the time series, showing the strong unsteadiness of the flow. The remainder of the cannula flow (around 96%) was returned through the cannula end hole, not shown in this plot

In both cases, the majority of the fluid (over 90%) was returned through the end hole. However, the distribution among the side holes differed between the two scenarios, shown in Fig. 4.

According to Fig. 4, in the tilted case, the holes on the narrow side (A, B and C) reinfused a larger fraction of fluid compared to the ones on the wide side, whereas the effect of cannula positioning on the out-of-plane holes was less relevant.

**Fig. 5:**
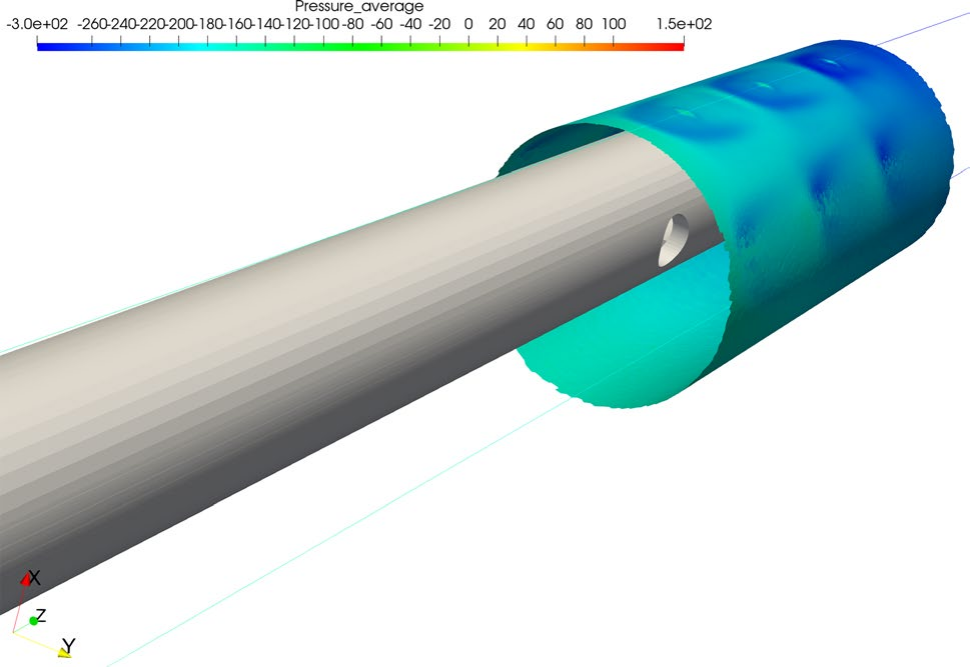
Pressure field in a cylindrical slice around the cannula for Case 2. The jets from the side holes on the narrow side were surrounded by low pressure areas, explaining why the flow rate through holes was higher compared to the wide side

Further insight into the near-wall dynamics is gained by looking at the pressure field on a cylindrical slice around the cannula, as depicted in Fig. 5. On the narrow side, the jets behave like obstacles to the co-flow, an aspect further explored in Sect. 3.3. An aspect of clinical relevance is the quantification of the forces acting on the walls of the cannula. In particular, both normal (pressure) forces and shear stress were integrated over the outer wall of the cannula and then decomposed along the three Cartesian axes, Table 2. Although small in magnitude, the force developing and acting in the *x* direction for the tilted case can be interpreted as acting to bring the cannula back to centered position. However, due to the inherent unsteadiness of the flow, the forces were characterized by large fluctuations. The contributions of pressure and viscous forces were singled out and decomposed along the coordinate axes. For the tilted case, the viscous forces in the *x*, *y* and *z* directions corresponded to 5%, 17% and 34%, respectively, of the total force.

**Table 2:** Time-averaged value of the forces (pressure + shear stress) integrated over the outer cannula walls

Case	$F_x (10^{-5}\,$N)	$F_y (10^{-5}\,$N)	$F_z (10^{-5}\,$N)
1	0.3	0.1	600
2	9	2	500

### Influence of the flow rate ratio

**Fig. 6:**
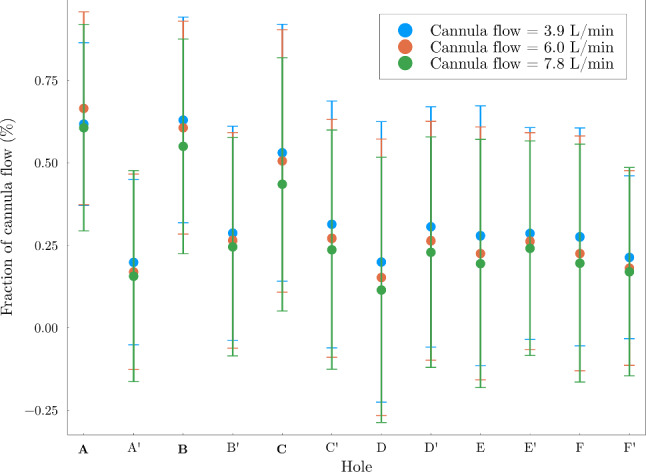
Fraction of reinfused flow rate from each side hole for Cases 3, 4 and 5

The fraction of fluid reinfused through the different side holes for different flow rate ratios (Cases 3 to 5) is shown in Fig. 6. The flow rate ratio played a minor role, with the large majority of the flow being reinfused through the end hole. Notably, the fractional flow rates through the side holes were rather insensitive to boundary conditions.

**Fig. 7:**
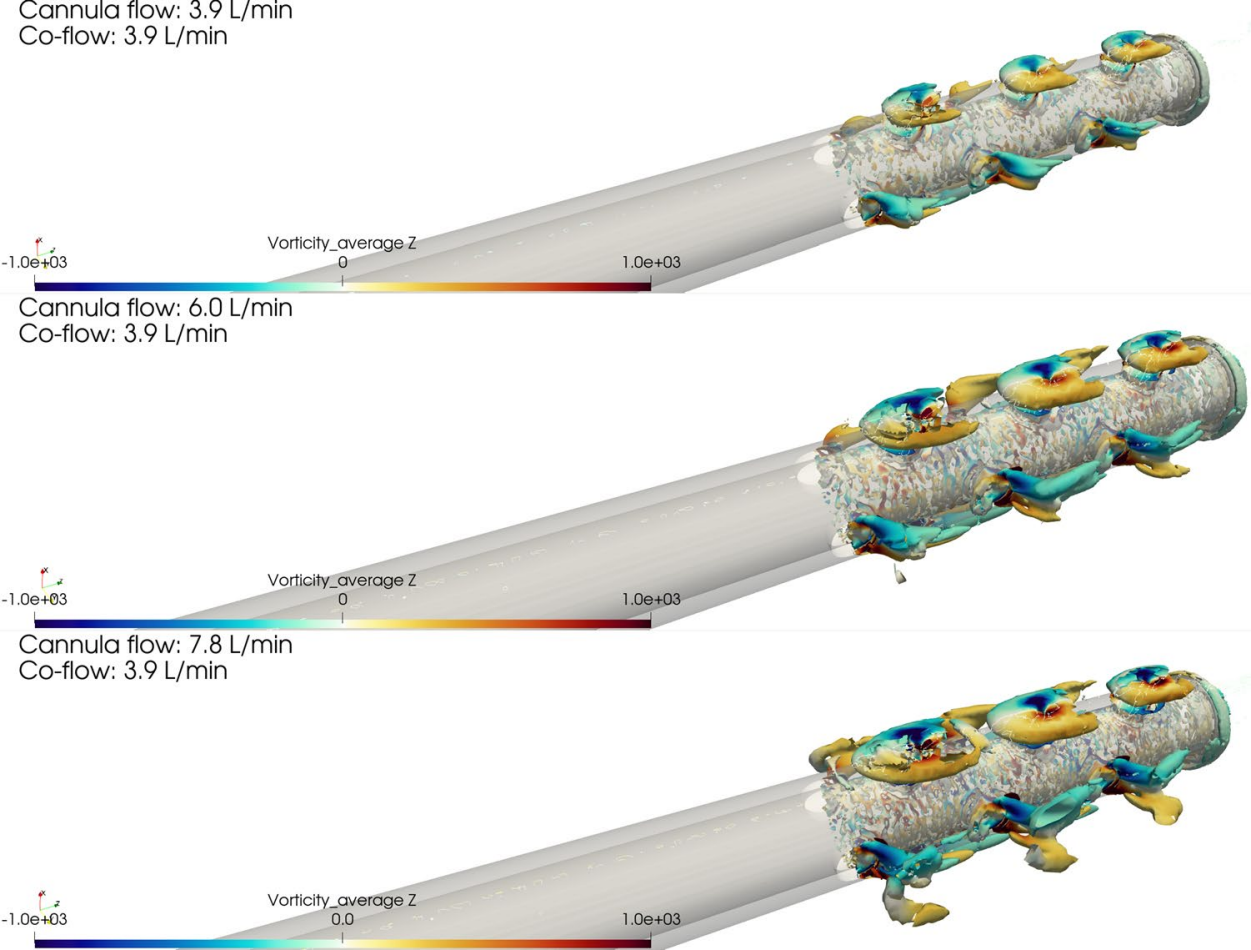
Q-criterion isosurfaces of the near-wall flow structures for Cases 3, 4 and 5 (the same threshold was used across all cases). The isosurfaces are colored by streamwise (*z* vorticity), showing that a pair of counter-rotating vortices was induced by the jet coming from the side holes

**Fig. 8:**
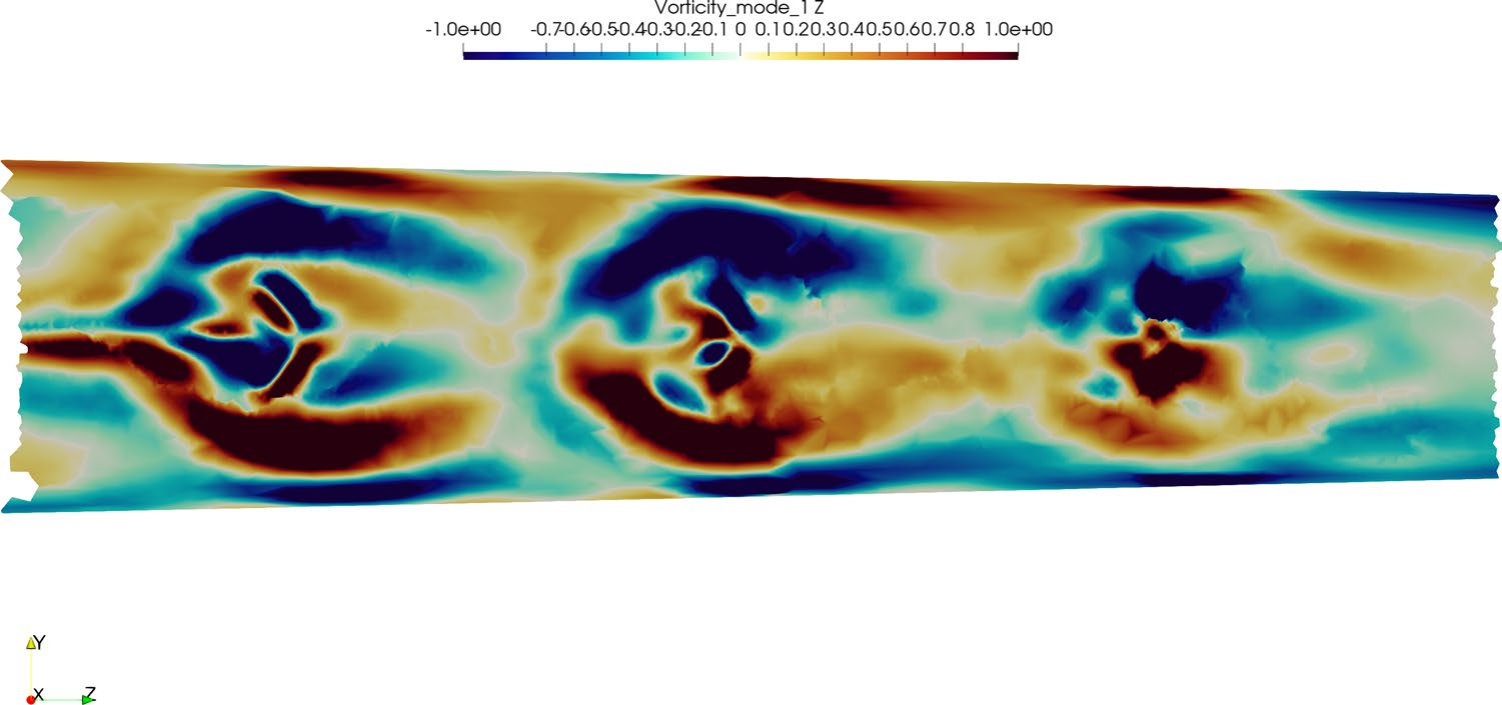
Axial (*z*) component of the vorticity computed from POD mode 1. The alternation of vortices with different rotation verse is clearly visible

As stated in Sect. 3.2, the jets coming out of the side holes on the narrow side formed structures obstructing the in-coming co-flow. A characteristic of this somewhat canonical flow is the development of a pair of counter-rotating vortices shedding with a periodic frequency, much like a Kármán vortex street (Krajnović 2011). This structures are depicted in Fig. 7 for the three different flow rate ratios with Q-criterion isosurfaces (using the same isovalue). The size of these structures increased with the flow rate ratio, also for the other side holes (A’, B’, etc.). A shedding of these vortices was observed on a plane perpendicular to the *x*-axis placed between the cannula and the vessel wall on the narrow side (i.e., a plane tangential to the cylinder depicted in Fig. 5), with a large scale flapping motion. This is also visible in Online Resource 3. To highlight the most energetic structures, a Proper Orthogonal Decomposition (POD) approach was applied to isolate the coherent vortical structures in the flow. From the first mode (excluding the mean, mode 0), the vorticity field was computed and shown in Fig. 8, sampled on the same plane as the animation shown in Online Resource 3. It is clear how closely the flow structures developing on the narrow side resembles the canonical configuration of a flow behind a cylindrical obstacle with the difference that the presence of three *obstacles* (i.e., the jets) disturbed the development of a vortex street; the discrete Fourier transform of the time coefficients of mode 1 showed a peak at 3 Hz, corresponding to a Strouhal number of 0.005 if the size of the cross-section of the jet and the bulk velocity of the co-flow are considered. However, it should be noted that the size of the jets and the local co-flow velocity showed variations both in space and time.

### Influence on hemolysis

Values of hemolysis index were compared between a centered and tilted case with cannula flow rate of 6.0 L/min and co-flow of 3.9 L/min using a Newtonian analog, as described in Sect. 2 and reported in Table 3.

**Table 3:** Hemolysis index values sampled at the domain outlet

Case	HI (%)
Centered, Newtonian	4.201E−5
Tilted, Newtonian	4.202E−5

**Fig. 9:**
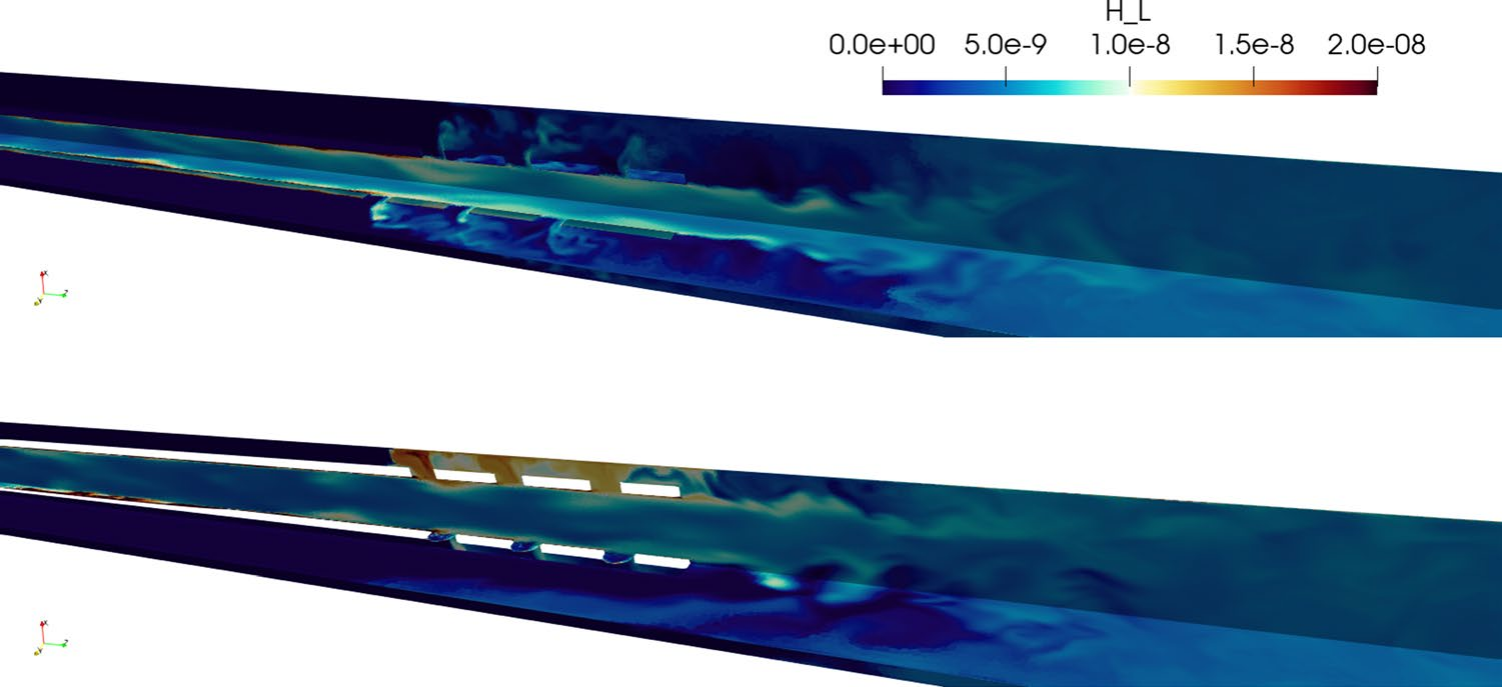
Instantaneous snapshots of hemolysis level on the centered case (top) and tilted (bottom). Local clusters of high hemolysis could be observed at the recirculation bubbles in the near-wall region. $H_L = H^{1/\beta }$

The values in Table 3 show that, at a global level, the change in cannula positioning did not introduce large variations in hemolysis values. However, the near-wall structures clearly affected the local blood damage risk, shown in Fig. 9. Given the small amount of fluid reinfused through the side holes (Fig. 6), this effect did not contribute to an overall larger hemolytic potential.

## Discussion and conclusions

The influence of positioning and flow rate ratio on a model of an ECMO return cannula was numerically assessed. The results showed that the flow field was similar to that of a canonical confined jet, with similar features compared to what was reported by Lemétayer et al. (2020, 2021). However, the presence of side holes introduced some noticeable differences. In the centered case, a non-pulsating backflow zone developed at the vessel wall (as opposed to the intermittent behavior reported by Lemétayer et al. (2020) for a blunt return cannula), with limited interaction between the side holes and the rest of the domain. The flow rate reinfused through the side holes, albeit strongly unsteady, was in all cases small compared to the end hole. An axial force was also observed directed as to bring the cannula further into the vessel.

The tilted case displayed interesting flow features. The backflow area on the wide side was more pronounced compared to the centered one, possibly leading to longer residence times. Moreover, on the narrow side, small vortical structures developed at each side hole, and confirmed by experiments. As shown in Fig. 5, the presence of the jets coming from the side holes created areas of low pressure, ultimately leading to consistently larger flow rates being reinfused from the holes on the narrow side compared to the wide. Relatively large shear stresses were also observed on the narrow side, which combined to the strength of the recirculation led to locally higher blood damage (Fig. 9), which however did not significantly impact the overall hemolytic potential of the cannula. The origin of the stresses acting in this area could be partially traced back to the development of pairs of counter-rotating vortices, due to the jets coming from the side holes behaving like obstacles to the flow. Interestingly enough, this phenomenon could be thought of as a *jet in crossflow* (Karagozian 2014), with three jets placed in a tandem. The Strouhal number was low compared to the classical jet in crossflow case. This could be related to the fact that the conditions were different from the canonical scenario (variable crossflow, unsteady, non-circular jets). Moreover, the frequency was computed from Fourier analysis of the POD time coefficients rather than from the local velocity signal (which was very sensitive to the location). Lemétayer et al. (2020) reported similar values of frequency associated to POD structures corresponding to the backflow of a blunt cannula for a cannula-to-vessel diameter ratio similar to what was investigated in the current study. The size of these flow structures was influenced by the flow rate ratios across Cases 3, 4 and 5, as depicted in Fig. 7, suggesting that blood damage levels might be higher at higher flow rates. Considering ECMO circuit components (pump, return cannula, draining cannula and connectors), the return cannula has been reported to be the second largest platelet activation promoter after the pump (Fuchs et al. 2018). Thus, motivating detailed flow studies to understand the underlying structures that may change blood trauma levels.

The fraction of fluid reinfused through each side hole was insensitive to the flow rate ratio; for a similar characterization sweep performed experimentally by Lemétayer et al. (2021) for a blunt cannula (i.e., without side holes), the overall mixing efficiency was also found to have no dependence on the flow rate ratio. Interestingly, the investigation by Lemétayer et al. (2021) showed that tilting the blunt cannula did not lead to much larger instantaneous or mean shear stress values compared to the centered case; however, the presence of side holes drastically changed the dynamics of the narrow side, leading to locally higher blood damage.

Another interesting phenomenon was the development of a radial force acting to re-center the cannula. While the magnitude of this force was very small (and highly unsteady) and no fluid–structure interaction (FSI) was modeled, this could have a more pronounced impact in the case of a flexible cannula. Using a simple cantilever beam model (i.e., considering the cannula bound at the inlet with a point load applied at the tip) with cross-section corresponding to that of the studied cannula and a Young modulus of 38 MPa (Ahmad et al. 2023), the maximum displacement was computed to be 0.7 mm. This warrants for further investigation, e.g., by using FSI models of flexible cannulae. This couples to the limitations of the current study, using a fully rigid model, with a larger wall thickness as compared to clinically used cannulae. A Newtonian model for blood was used, which may introduce uncertainties away from the cannula and where stagnant areas are present, in terms of induced stress.

In conclusion, the results showed that a tilted cannula reinfused a larger amount of fluid through the side holes on the narrow side compared to the wide side, but the fraction of reinfused fluid is not influenced by the flow rate ratio. Small recirculating vortical structures developed on the narrow side, potentially leading to higher blood damage compared to the centered case. This effect could be partially mitigated by the presence of a radial force trying to bring the cannula back toward the center. The reinfused fluid coming from the A, B and C side holes behaves like an obstacle to the co-flow, leading to the development of counter-rotating vortex pairs with a flapping behavior, contributing to a dynamic stress field which impacted local values of hemolysis, yet not contributing to significantly higher levels of RBC damage over the whole domain.

### Supplementary Information

Below is the link to the electronic supplementary material.

**Supplementary information.** Online resource 1 (document): Details of the simulation setup, including meshing, discretization, and validation with an experimental campaign. (pdf 3276KB)

**Supplementary information.** Online resource 2 (video): Animation of the axial component of the flow field in a longitudinal cross section. (pdf 13849KB)

**Supplementary information.** Online resource 3 (video): Animation of the axial component of the flow field on a cut plane in the narrow side of the cannula, between the cannula wall and the vessel wall. (pdf 2971KB)

## Data Availability

The produced datasets are available upon reasonable request.
