# Supplementary material for: Effect of flow rate ratio and positioning on a lighthouse tip ECMO return cannula
Source: Biomech Model Mechanobiol. 2023 Jul 16;22(6):1891–9. doi: 10.1007/s10237-023-01741-2 (PMC10613146; doi:10.1007/s10237-023-01741-2)
Supplement: Supplementary file 1 — Supplementary information. Online resource 1 (document): Details of the simulation setup, including meshing, discretization, and validation with an experimental campaign. (pdf 3276KB) [file 10237_2023_1741_MOESM1_ESM.pdf]

Supplemental Material 1: Meshing & validation

A snapshot of the mesh at the reinfusion area is provided in Figure S1.1 Five prism layers were grown from the walls to resolve the boundary layer. Different refinements were applied to resolve highly dynamic areas: both around the holes and downstream of the cannula tip, a volumetric refinement was applied to prescribe a honeycomb structured-like mesh topology in two cylindrical regions. Moreover, a refinement was prescribed to account for jet spreading in a conical wake shape. Refinements were described in terms of percentage of a *base size*. The quality of the mesh was also evaluated in terms of a metric recovered from velocity gradients. A local flow length scale was computed by dividing the velocity magnitude by the strain rate modulus, thus obtaining a "gradient length scale". This length was divided by the cell size (computed as the cubic root of the cell volume), thus obtaining the number of cells per length scale. For the centered case, this is shown in Figure S1.2. The color bar in Fig. S1.2 was capped to 1 to highlight areas where the cell size was larger than the length scale, which represents a small portion of the domain of interest. In most of the computational domain, the cell quality metric was > 2.

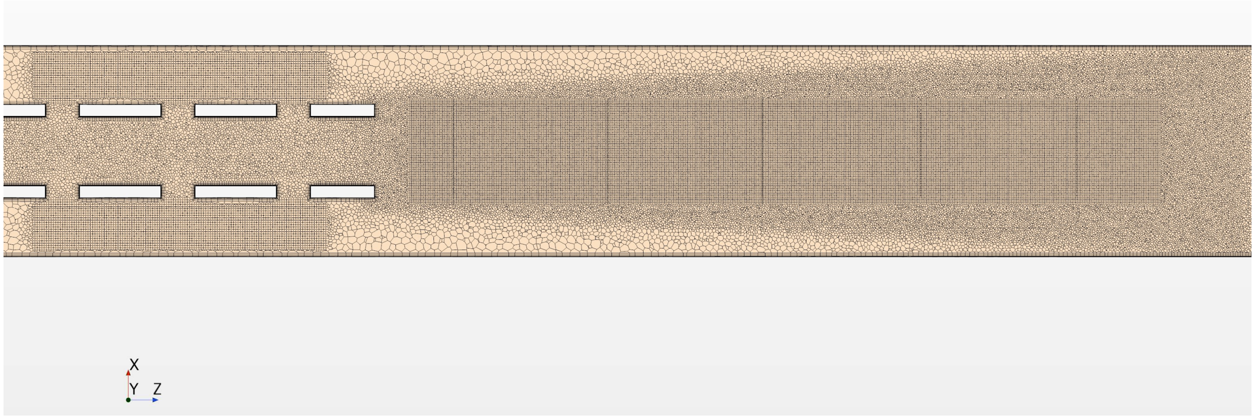

Figure S1.1: computational mesh for the centered case

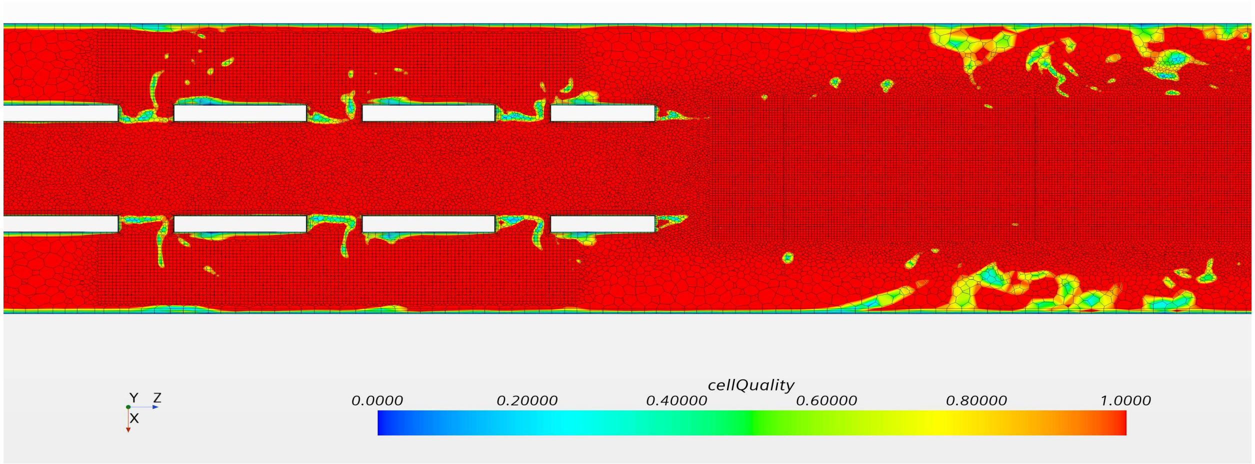

Figure S1.2: Cell quality metric. Be slightly more descriptive, what is shown.

Given that implicit LES were used, stricter requirements are imposed on the timestep. Figure S1.3 below shows a snapshots of instantaneous convective Courant number, with the Courant number being less than 1 almost throughout the domain. Moreover, an appropriate number of outer correctors was used to ensure stability.

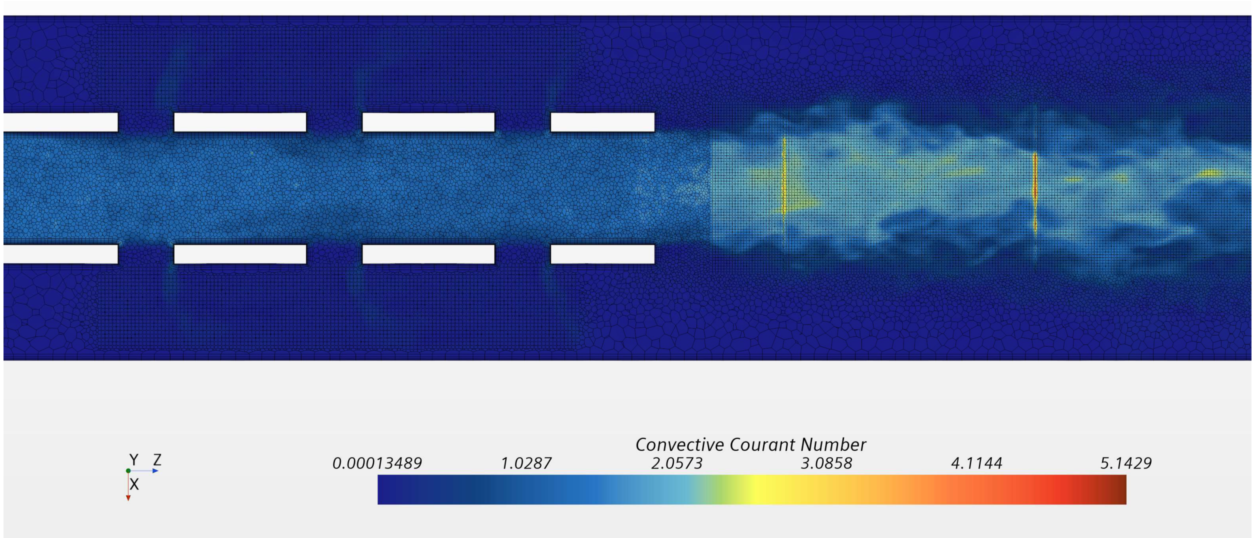

Figure S1.3: Instant convective Courant number displayed for the midplane of the computational domain.

Regarding the tilted case, the results of the mesh convergence study consisting of three meshes of 6.7M, 10.7M and 11.9M cells are shown in Figure S1.4. The magnitude of velocity extracted at lines going through the side holes A, B and C and downstream of the tip showed good agreement. Compared to experimental data, differences were observed at distances further downstream of the cannula tip. To assess potential influence of numerical dissipation, implicit versus explicit LES (incl. subgrid-scale modeling) was compared, Fig. S1.5. For the furthermost downstream velocity lines, the use of a WALE subgrid-scale model showed better agreement with the experimental data, although still displaying differences at the furthermost line (30 mm), Fig. S1.6. This highlights the care needed when simulating these highly unsteady flows.

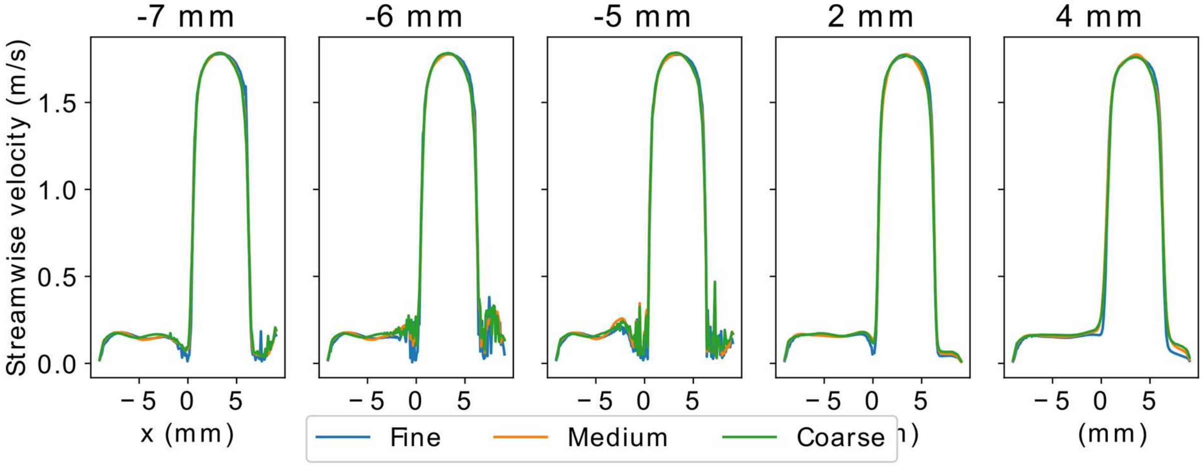

Figure S1.4: Mesh convergence study for the tilted case comparing three different meshes consisting of 6.7M, 10.7M and 11.9M cells respectively.

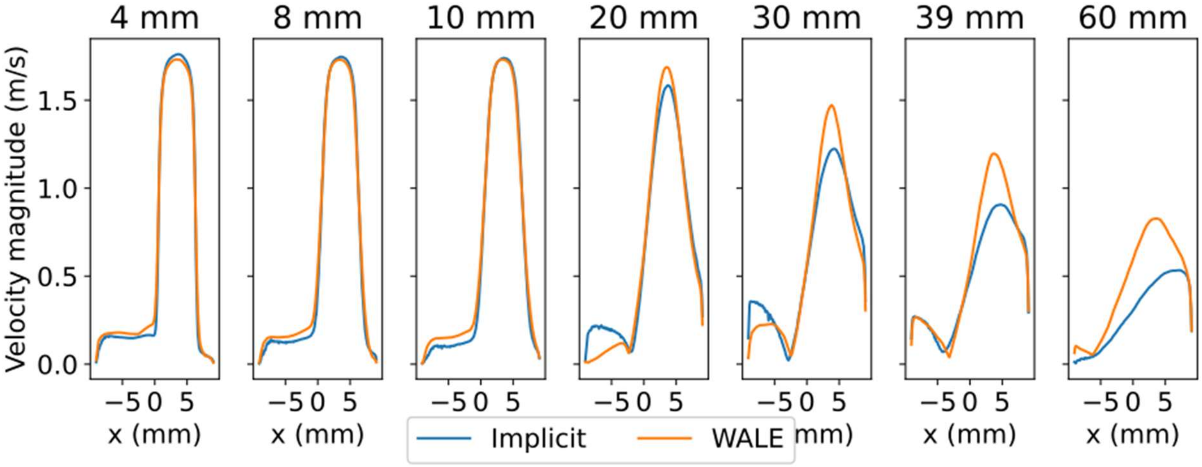

Figure S1.5: Comparison between WALE and implicit LES, showing that results are reliable in the proximity of the cannula tip, whereas WALE agreed better with experimental results further downstream.

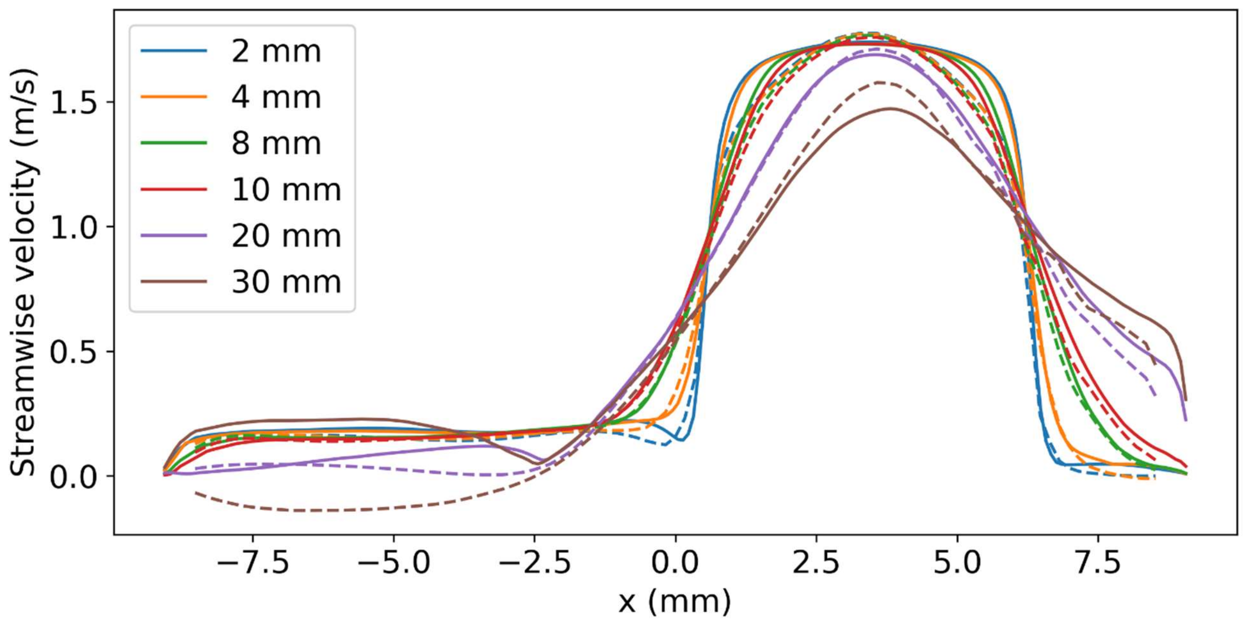

Figure S1.6: Comparison between experimental data and numerical data obtained with a WALE SGS (solid line numerical, dashed line experimental).
